# Effect of Targeted Behavioral Science Messages on COVID-19 Vaccination Registration Among Employees of a Large Health System: A Randomized Trial

**DOI:** 10.1001/jamanetworkopen.2021.18702

**Published:** 2021-07-28

**Authors:** Henri C. Santos, Amir Goren, Christopher F. Chabris, Michelle N. Meyer

**Affiliations:** 1 Behavioral Insights Team, Steele Institute for Health Innovation, Geisinger Health System, Danville, Pennsylvania; 2 Autism and Developmental Medicine Institute, Geisinger Health System, Lewisburg, Pennsylvania; 3 Center for Translational Bioethics and Health Care Policy, Geisinger Health System, Danville, Pennsylvania

## Abstract

This randomized trial evaluates whether individually addressed emails designed with behaviorally informed features increase COVID-19 vaccination rates.

## Introduction

The first opportunities to field test interventions to increase COVID-19 vaccination were among health care workers (HCWs), who were among the first to be offered COVID-19 vaccines. After 1 large Pennsylvania health system sent 36 vaccine-related mass emails to employees over 5 weeks (eAppendix in Supplement 1), 9723 of 23 700 HCWs (41%) had still not scheduled their vaccination. We sought to determine whether individually addressed emails designed with behaviorally informed features^1,2,3,4,5^ could increase vaccination registration compared with a delayed control group.

## Methods

This project followed the Consolidated Standards of Reporting Trials (CONSORT) reporting guideline. The Geisinger institutional review board determined that this health care operations project did not constitute human participants research and that a follow-up research analysis was exempt from review or the requirement for informed consent under 45 CFR §46.104(d)(4)(iii). The trial protocol is available in Supplement 2.

In this randomized trial, we assigned 9723 employees (eFigure in Supplement 1) who had not scheduled a COVID-19 vaccination to a delayed control condition (3241 [33%] randomized; 3179 [33%] received intervention) or to receive 1 of 2 individually addressed emails with 3 components. Both emails explained that Pennsylvania would soon expand vaccine eligibility beyond HCWs, reducing employees’ access to appointments, and encouraged them to schedule an appointment. The 6482 employees in these intervention groups were assigned to receive an email that framed the decision to be vaccinated either by noting that many US residents and fellow employees had chosen to be vaccinated, ie, social norms^2^ (3241 [33%] assigned; 3198 [33%] received intervention) or by favorably juxtaposing the vaccine’s risks with those of COVID-19, ie, reframing risks^3^ (3241 [33%] assigned; 3190 [33%] received intervention). Both emails asked employees to make an active choice^4^ to receive a vaccine (hyperlinked to a scheduling portal) or not (hyperlinked to a survey soliciting their primary reason for declining). Employees in the delayed condition were randomly assigned to receive 1 of these emails (social norms: 1589 [50%]; reframing risks: 1589 [50%]) 3 days later. The primary outcome was registration on the vaccination scheduling portal during the 3 days after the first emails were sent.

Random assignment (with the randomizr package) and logistic regression analyses were conducted using R version 4.0.2 (R Project for Statistical Computing). For all analyses, odds ratios (ORs) from logistic regressions were calculated, along with asymptotic 95% CIs; 2-tailed *P* < .05 was used to determine statistical significance. Detailed methods appear in the eAppendix in Supplement 1.

## Results

**Figure 1.**
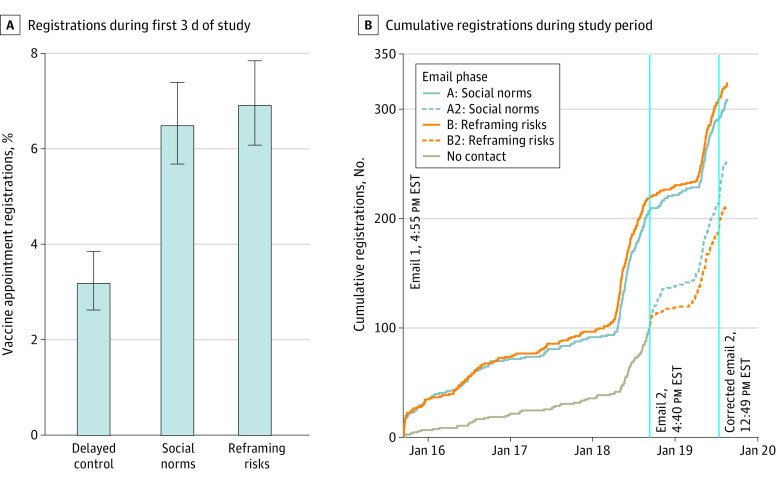
Vaccine Appointment Registrations by Email Condition Panel A shows registrations during the first 3 days of the study (January 15, 2021, 4:55 pm, to January 18, 2021, 4:40 pm). Error bars represent asymptotic 95% CIs. Panel B shows cumulative registrations over time. Email 2 and corrected email 2 were only sent to participants in A2 and B2. The lines for A2 and B2 represent registrations made by the delayed control group after they were sent the social norms and reframing risks emails, respectively. Data collection ended on January 19, 2020, at 3:19 pm, when the provided link no longer forwarded employees to the employee registration portal.

**Figure 2.**
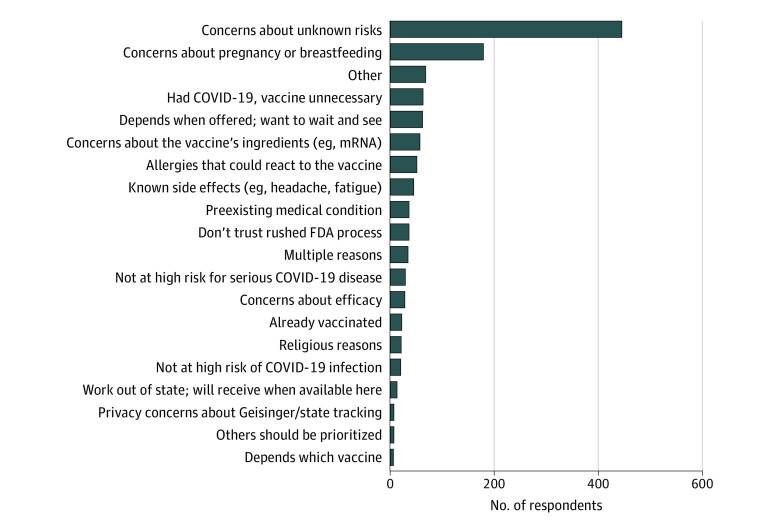
Main Reasons for Declining to Schedule Vaccination Overall, 239 of 1229 respondents (19%) who declined to receive the vaccine provided free-text reasons rather than choose 1 of the reasons listed in the survey. Most of these were recategorized into existing reasons or 1 of 4 new categories: multiple reasons, already vaccinated, religious reasons, and others should be prioritized. The remaining respondents were left in the other category.

The overall employee population of 23 700 HCWs comprised 17 362 (73%) women and 21 168 (89%) White employees, with a mean age of 43 years. Of the 9723 targeted employees, 9457 (97%) had valid email addresses. Both emails (ie, social norms and reframing risks) led to more registrations in the first 3 days than the delayed condition (delayed control group: percentage of participants registering: 3.17%; 95% CI, 2.62%-3.85%; social norms: percentage of participants registering, 6.47%; 95% CI, 5.67%-7.38%; OR, 2.11; 95% CI, 1.65-2.69; *P* < .001; reframing risks: percentage of participants registering, 6.90%; 95% CI, 6.07%-7.83%; OR, 2.26; 95% CI, 1.77-2.87; *P* < .001) (Figure 1). There was no significant difference in registrations between the 2 email conditions (OR, 1.07; 95% CI, 0.88-1.30; *P* = .50). Among the 1229 HCWs who declined to register and then completed the survey, the most common reasons were unknown vaccine risks (430 [35%]) and pregnancy-related concerns (165 [13%]) (Figure 2).

## Discussion

During the 3-day study period, an individual email nudge caused more than twice as many HCWs to register for a COVID-19 vaccination compared with HCWs in the control condition, with no significant difference between the 2 emails. A limitation of this trial is that due to the imminent closure of employee-only vaccination clinics, we could only delay the intervention in the control group by 3 days. Moreover, by choosing to compare 2 behaviorally informed emails, we are unable to exclude the possibility that a plain reminder might have had the same effect. Furthermore, we could not measure actual vaccination, as appointment slots were unexpectedly unavailable for many who registered for one.

Given the large volume of previous COVID-19 vaccine promotion to HCWs, it may seem counterintuitive that a single additional reminder could increase vaccination by late adopters. However, competing demands on attention, behavioral inertia, and unwieldy processes that make it hard to follow through on intentions likely conspire to make a single, timely, targeted reminder helpful.^2,3,4,5^ The emails’ behavioral features—active choice, appeal to authority, and emphases on scarcity, social norms, and risk recalibration (eAppendix in Supplement 1)—may have contributed to their effect. The 3.17% absolute increase in vaccination appointments we observed is larger than many real-world nudges^6^ and may be greater among recipients who are less hesitant about receiving the vaccine. Sending targeted emails, patient portal messages, or text messages designed with behavioral science is inexpensive, scalable, and easily implemented and could be an effective way to encourage vaccination by HCWs and the general public.

## Notes

Supplement 1. **eAppendix. **Supplementary Methods **eFigure.** Study Flowchart eReferences.

Supplement 2. Trial Protocol

Supplement 3. Data Sharing Statement

## References

[1] Milkman KL, Patel MS, Gandhi L, . A megastudy of text-based nudges encouraging patients to get vaccinated at an upcoming doctor’s appointment. Proc Natl Acad Sci U S A. 2021;118(20):e2101165118. doi:10.1073/pnas.2101165118 33926993 PMC8157982

[2] Moehring A, Collis A, Garimella K, . Surfacing norms to increase vaccine acceptance. psyArXiv. Preprint updated March 19, 2021. doi:10.31234/osf.io/srv6t

[3] Tversky A, Kahneman D. The framing of decisions and the psychology of choice. Science. 1981;211(4481):453-458. doi:10.1126/science.7455683 7455683

[4] Patel MS, Volpp KG, Small DS, . Using active choice within the electronic health record to increase influenza vaccination rates. J Gen Intern Med. 2017;32(7):790-795. doi:10.1007/s11606-017-4046-6 28337690 PMC5481246

[5] Bakr O, Afsar-Manesh N, Raja N, . Application of behavioral economics principles improves participation in mailed outreach for colorectal cancer screening. Clin Transl Gastroenterol. 2020;11(1):e00115. doi:10.14309/ctg.0000000000000115 31972609 PMC7056051

[6] DellaVigna S, Linos E. RCTs to scale: comprehensive evidence from two nudge units. NBER Working Paper. July 28, 2020. Accessed June 22, 2021. https://papers.ssrn.com/sol3/papers.cfm?abstract_id=3661086

