# Supplementary material for: Effect of Targeted Behavioral Science Messages on COVID-19 Vaccination Registration Among Employees of a Large Health System: A Randomized Trial
Source: JAMA Netw Open. 2021 Jul 28;4(7):e2118702. doi: 10.1001/jamanetworkopen.2021.18702 (PMC8319759; doi:10.1001/jamanetworkopen.2021.18702)
Supplement: Supplement 1. — eAppendix. Supplementary Methods eFigure. Study Flowchart eReferences. [file jamanetwopen-e2118702-s001.pdf]

Supplemental Online Content

Santos HC, Goren A, Chabris CF, Meyer MN. Effect of targeted behavioral science messages on COVID-19 vaccination registration among employees of a large health system: a randomized trial. *JAMA Netw Open.* 2021;4(7):e2118702. doi:10.1001/jamanetworkopen.2021.18702

eAppendix. Supplementary Methods

eFigure. Study Flowchart

eReferences.

This supplemental material has been provided by the authors to give readers additional information about their work.

© 2021 Santos HC et al. *JAMA Network Open.*

eAppendix. Supplementary Methods

The data, materials, and reproducible code used in this study have been deposited to OSF: https://osf.io/qg5m6/.

Participants

Data were collected from Geisinger Health System employees, most of whom work remotely or in-person in central and northeastern Pennsylvania (location was not a criterion for eligibility). Sample size was determined by the number of eligible employees (had our study not occurred, all would have received one email from the health system promoting vaccination). Employees have received at least 36 mass emails from the initial announcement of a vaccination plan to the start of the intervention (with two more sent after the intervention), which are detailed in the table below.

Supplement Table 1: Mass emails from December 11 to January

Administrative Email	Daily Online Newsletter	Description	Link to Scheduling Portal in Email	Addressed by Name
12/11		Announcement from CEO on vaccination plan		
	12/11	Link to CEO announcement		
	12/14	Link to updated FAQ about vaccination plan		
12/15		News story: Vaccines have arrived		
12/15		Virtual town hall announcement: Vaccine and registration information		
	12/15	News story: Vaccines arrive		
	12/16	News story: First employees vaccinated		
12/17		Stay healthy message; link to vaccine FAQ		
	12/17	Link to information on where to get vaccine		
12/18		Link to vaccination plan		X
	12/18	Link to updated vaccine FAQ, Link to personal testimonial on vaccination		
	12/21	Subject line on not spreading COVID-19 during the holidays; link to story about light at the end of the tunnel		
12/22		Vaccine update: appointments available	X	
12/22		Summary of vaccine news; notice of available appointments		
	12/22	Link to an announcement about appointments being available; link to an FAQ on scheduling		
12/23		Announcement: new appointments available at specific site	X	
	12/23	Link to announcement: new appointments available at specific site		
12/24		Announcement: new appointments available at specific site	X	
	12/24	Links to announcements, vaccine safety information		
12/27		Announcement: vaccine available to all employees	X	
	12/28	Link to announcement: vaccine available to all employees		

© 2021 Santos HC et al. *JAMA Network Open.*

	12/29	Link to personal testimonial on vaccination; link to podcast on vaccines		
	12/30	Link to FAQ; link to personal testimonial on vaccination		
12/30		Announcement: appointments available in multiple sites	X	X
	12/31	Fear COVID not the vaccine subject line; link to personal testimonial on vaccination		
	01/04	Link to video of CEO administering vaccines; link to podcast on vaccines		
	01/05	Link to vaccine safety FAQ		
	01/06	News story: employees received second doses; link to podcast on vaccines		
01/07		Vaccine FAQ	X	
	01/07	Link to vaccine mythbusting; link to personal testimonial on vaccination; link to vaccine information		
01/08		Schedule your vaccine message from leadership		X
	01/08	Link to vaccine mythbusting; link to vaccine informational video; links to news stories and information about the vaccines		
	01/11	Link to virtual press conference; links to news stories and information about the vaccines		
	01/12	Reminder to schedule appointment; link to personal testimonial on vaccination		
	01/13	Link to video on vaccine side-effects; link to story on using PTO for vaccine side-effects, link to article about vaccine safety		
	01/14	Link to personal testimonials on vaccination		
01/15		Link to announcement asking employees to get vaccinated; link to personal testimonials on vaccination		X
	01/15	News story: 20,000 vaccinated update; link to personal testimonial on vaccination		
	01/18	Links to announcements and news stories about the vaccines		
	01/19	Link to vaccine mythbusting, links to announcements and news stories about the vaccines		

We used a 1 x 3 factorial design, and Dr. Santos randomly assigned participants with a random number generator in R providing the seed value and the randomizr package, which evenly distributed participants into 3 groups. Geisinger's Human Resources Department only provided individual information on job roles, and the balance between groups is shown in the table below. Participants were enrolled by virtue of being sent the email. Emails were sent with Microsoft Dynamics. Participants did not know to which group they were assigned (for the purposes of reporting masking procedures, there were no providers involved in the intervention). From an original list of 9,723 employees, 157 were not able to be reached with the email address on record, and 1 research team member was removed because they were accidentally included in the list (see the CONSORT flowchart below).

Supplement Table 2: Distribution by Job Category

Job Category	Percentage		
	Delayed Control	Social Norms	Reframing Risks
Academic	0.63	0.94	0.72
Facility Operations	9.85	10.85	10.34
Finance	4.09	4.88	5.17
General Administration	1.86	2.25	1.94
Health Services	20.58	19.48	19.84
Human Resources	0.82	0.66	0.60
Information Services	8.40	8.07	7.27
Insurance Operations	4.34	4.63	4.42
Learners	0.79	0.97	0.97
Nursing	30.21	28.30	29.62
Provider	5.82	6.16	5.55
Research	1.42	1.53	1.19
Support Services	11.20	11.29	12.32

Materials

Both emails were written in the form of a personal message from a medical expert and authority figure on COVID-19 in the health system (the Division Chief of Infectious Diseases). The first paragraph of both emails emphasized the upcoming scarcity of the vaccines, and the second paragraph encouraged employees to make an active choice of whether to get the vaccine or not. The social norms email used an action-oriented subject line: "Indicate your COVID-19 vaccine plan." The reframing risk email used a matter-of-fact subject line: "A message from Dr. Stan Martin."

The online survey (reached by clicking a link in the email to decline to schedule a vaccination appointment) presented the following question: "What is the main reason you're declining to receive an FDA-authorized COVID-19 vaccine? (Your responses are anonymous and cannot be linked to your name or email.)" It then presented 15 possible options: (1) Concerns about known side effects (e.g., headache, fatigue); (2) Concerns about unknown risks; (3) I don't feel I need to be vaccinated; I'm not at high risk for infection; (4) I don't feel I need to be vaccinated; I'm not at high risk for serious complications from COVID if I am infected; (5) It depends on when it's offered to me; I'd like to wait to take it until I see how it goes with others; (6) It depends on which vaccine I'm offered; (7) I've had COVID so I don't think I need a vaccine; (8) Privacy concerns about Geisinger or state government tracking; (9) I don't trust the FDA approval process; (10) I am pregnant, breastfeeding or planning to become pregnant; (11) I have a preexisting medical condition that would prevent me from getting the vaccine; (12) I have allergies that could react to the vaccine; (12) Concerns about the vaccine's ingredients (e.g., mRNA); (13) Concerns about the vaccine's efficacy; (14) I work out of state; I plan to receive a vaccine when one is available to me in my state; (15) Other [free response item].

After respondents selected their main reason, an explanation was displayed that addressed that particular concern. These explanations were largely drawn from the health system's online materials on the Pfizer/BioNTech and Moderna vaccines. After reading this explanation, respondents were given a second opportunity to make an appointment, but only three clicked on the registration site link.

Intervention Methods

The first set of emails was sent to employees in the social norms and reframing risks groups at 16:55 EST, January 15, 2021 (see Email Versions A and B in the Annotated Materials, below). An analysis of the primary outcome was planned for January 18, 2021, but the data was not able to be reliably gathered at that time. Without any reliable data on the performance of the two groups from which to choose a clearly superior email to send to the delayed control group, we decided to evenly randomize that group's members to the two email versions (see Email Versions A2 and B2, below). Of this group, 63 were not able to be reached with the email address on record. This second set of the two treatment emails was sent to the delayed control group at 16:40 EST, January 18, 2021. A

corrected version of each of these emails was sent at 12:49 EST, January 19, mentioning a change in state guidelines on the next stage of vaccine distribution (see Email Versions A2—Corrected and B2—Corrected, below). The correction did not change any information about the distribution plan for employees, registration method, or upcoming deadline.

The primary outcome was the number of employees who registered for a vaccine appointment during the first three days (i.e., from January 15 to 18), but additional data was gathered until 15:19 EST on January 19, which was when our provided link no longer forwarded employees to the employee appointment system closed. Vaccine registration involves visiting the online scheduling portal on Quickbase, where employees first entered their employee ID and email address, then on a second screen completed an attestation form and screening questionnaire (11 items), acknowledged reading FAQs, and clicked a button labelled submit. Pressing submit on this second page recorded the registration in Quickbase and constitute a registration for purposes of our results, regardless of eligibility for vaccination or the availability of vaccination appointments. Merely entering the employee's ID and email address and proceeding to the second page, without completing these further steps, did not constitute a vaccination registration. Data were initially delivered to the authors on February 3, 2021. Additional data were delivered on May 13, 2021, to enable the research team to more precisely determine how far in the registration process each employee had gone.

Analysis Methods

In our ClinicalTrials.gov record, we preregistered that the study would examine vaccination registration data after two days, a plan we had created on January 15. When it became apparent that the delayed control group would not be emailed until the third day, and without having received any data, we changed the primary outcome period to three days. Additional "engagement metric" data—email opens and link clicks—were also obtained, and will be uploaded on the Open Science Framework (institutional approval is pending). Email opens (depending on the platform, this was recorded by either opening the email or viewing the preview pane) and clicks were reported through Microsoft Dynamics. We preregistered these as secondary outcomes, but with no hypotheses, and they were not analyzed as outcomes in this study.

For all analyses, odds ratios (ORs) from logistic regressions were calculated, along with asymptotic 95% confidence intervals (CIs); two-tailed p-values < 0.05 were used to determine statistical significance. Two logistic regression models were run in parallel. One compared the email conditions against the delayed control group (reference group), and the other compared the social norms email against the reframing risks email (reference group).

There were no harms or unintended effects reported.

Annotated Materials

Email Version A: Social Proof

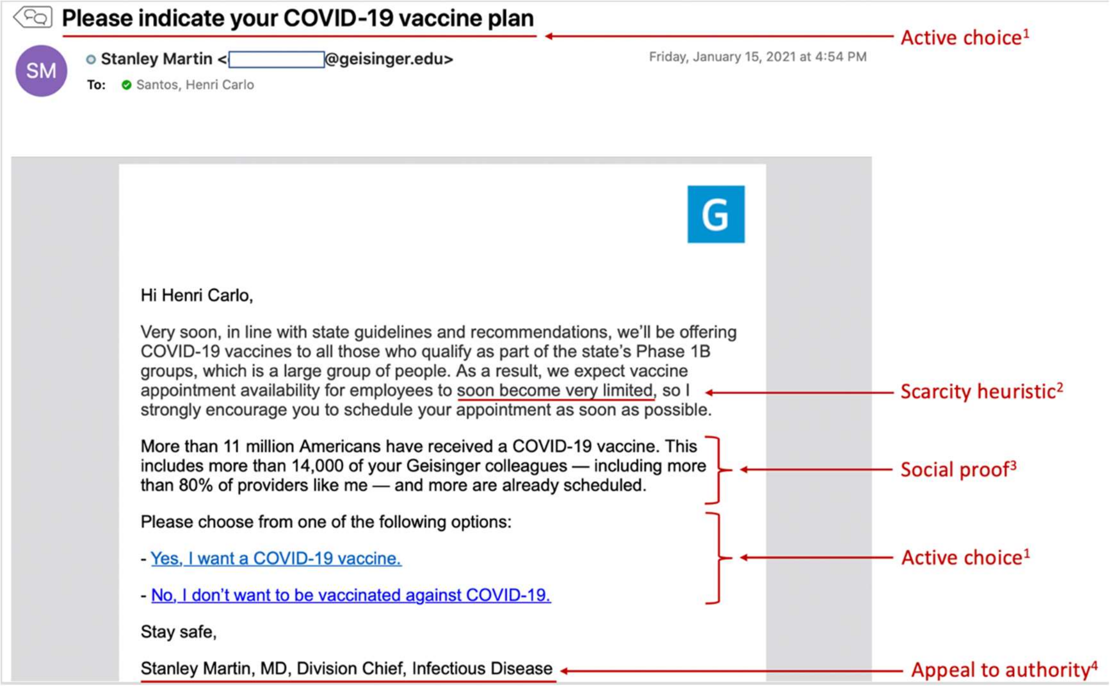

Email Version B: Reframing Risk

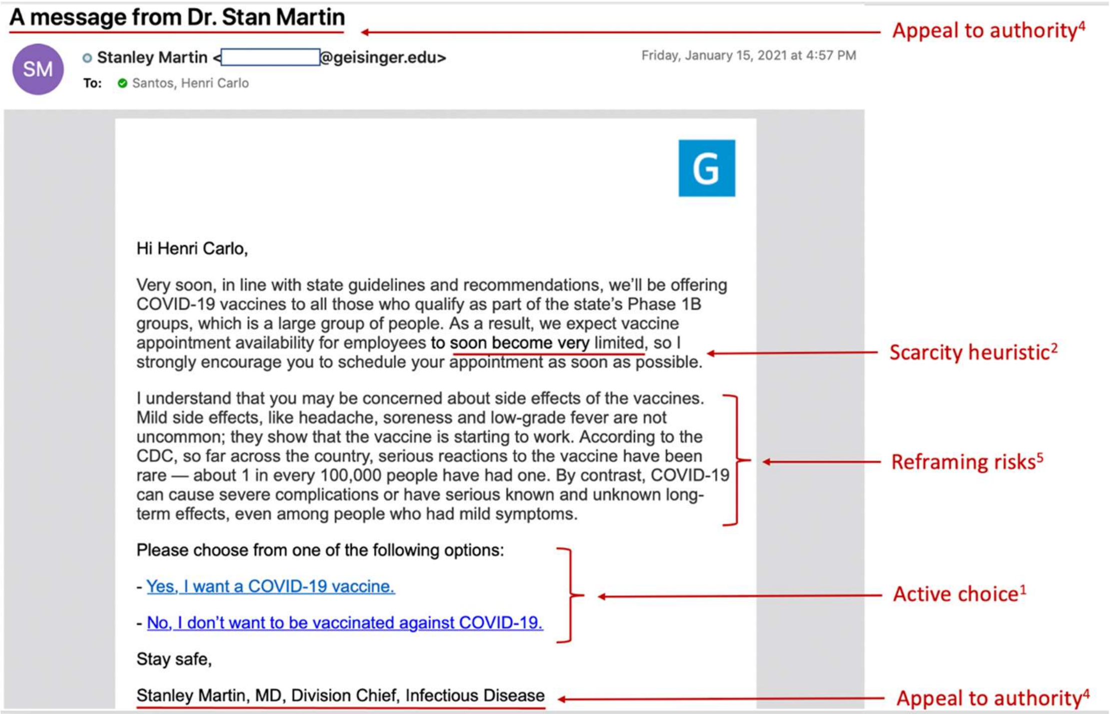

© 2021 Santos HC et al. *JAMA Network Open.*

Email Version A2: Social Proof (delayed control)

Please indicate your COVID-19 vaccine plan: deadline tomorrow

SM Stanley Martin <@geisinger.edu> Monday, January 18, 2021 at 4:39 PM

To: Santos, Henri Carlo

G

Hi Henri Carlo,

Tomorrow is the final day you can easily receive a COVID-19 vaccine as an employee. Starting Wednesday, in line with state guidelines and recommendations, we'll begin offering COVID-19 vaccines to all those who qualify as part of the state's Phase1B groups, a large group of people. As a result, we expect vaccine appointment availability for employees to soon become very limited, so I strongly encourage you to schedule your appointment as soon as possible.

New text added to Email Version A

More than 11 million Americans have received a COVID-19 vaccine. This includes more than 14,000 of your Geisinger colleagues—including more than 80% of providers like me—and more are already scheduled.

Please choose from one of the following options:

- Yes, I want a COVID-19 vaccine.
- No, I don't want to be vaccinated against COVID-19.

Stay safe,

Stanley Martin, MD, Division Chief, Infectious Disease

Email Version B2: Reframing Risk (delayed control)

A message from Dr. Stan Martin: deadline tomorrow

SM Stanley Martin <geisinger.edu> Monday, January 18, 2021 at 4:42 PM

To: Santos, Henri Carlo

G

Hi Henri Carlo,

Tomorrow is the final day you can easily receive a COVID-19 vaccine as an employee. Starting Wednesday, in line with state guidelines and recommendations, we'll begin offering COVID-19 vaccines to all those who qualify as part of the state's Phase1B groups, a large group of people. As a result, we expect vaccine appointment availability for employees to soon become very limited, so I strongly encourage you to schedule your appointment as soon as possible.

New text added to Email Version B

I understand that you may be concerned about side effects of the vaccines. Mild side effects, like headache, soreness and low-grade fever are not uncommon; they show that the vaccine is starting to work. According to the CDC, so far across the country, serious reactions have been rare — about one in every 100,000 people have had one. By contrast, COVID-19 can cause severe complications or have serious known and unknown long-term effects, even among people who had mild symptoms.

Please choose from one of the following options:

- Yes, I want a COVID-19 vaccine.
- No, I don't want to be vaccinated against COVID-19.

Stay safe,

Stanley Martin, MD, Division Chief, Infectious Disease

© 2021 Santos HC et al. *JAMA Network Open.*

Email Version A2—Corrected: Social Proof (delayed control)

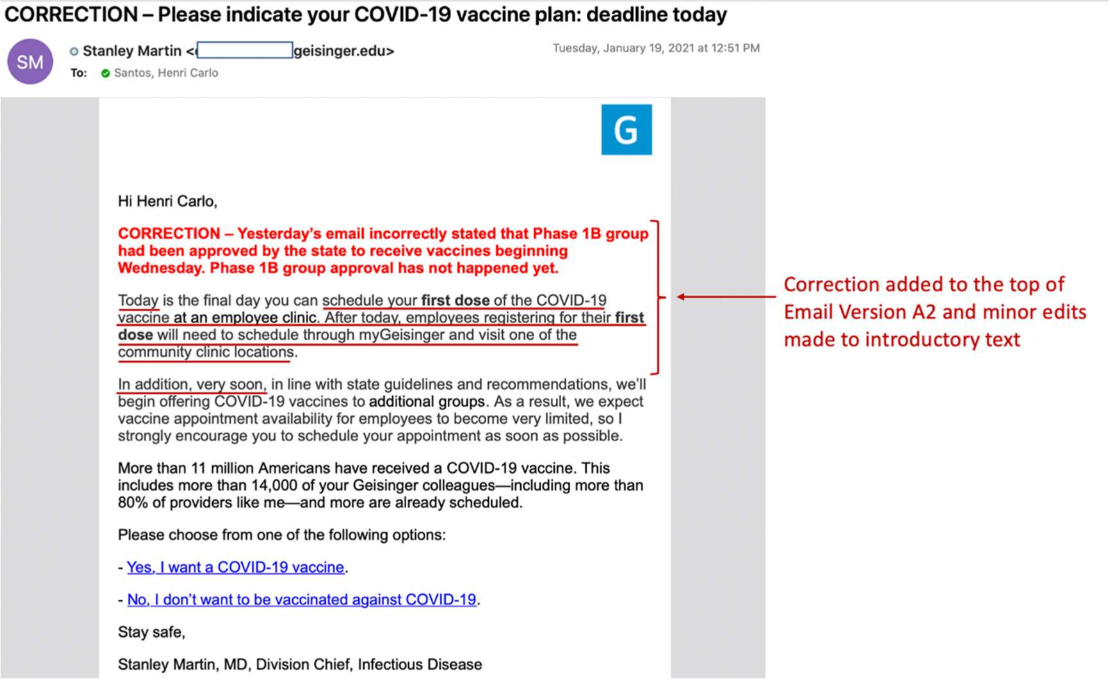

CORRECTION – Please indicate your COVID-19 vaccine plan: deadline today

SM Stanley Martin <[illegible]geisinger.edu> Tuesday, January 19, 2021 at 12:51 PM
To: Santos, Henri Carlo

G

Hi Henri Carlo,

CORRECTION – Yesterday's email incorrectly stated that Phase 1B group had been approved by the state to receive vaccines beginning Wednesday. Phase 1B group approval has not happened yet.

Today is the final day you can schedule your **first dose** of the COVID-19 vaccine **at an employee clinic**. After today, employees registering for their **first dose** will need to schedule through myGeisinger and visit one of the community clinic locations.

In addition, very soon, in line with state guidelines and recommendations, we'll begin offering COVID-19 vaccines to **additional groups**. As a result, we expect vaccine appointment availability for employees to become very limited, so I strongly encourage you to schedule your appointment as soon as possible.

More than 11 million Americans have received a COVID-19 vaccine. This includes more than 14,000 of your Geisinger colleagues—including more than 80% of providers like me—and more are already scheduled.

Please choose from one of the following options:

- Yes, I want a COVID-19 vaccine.

- No, I don't want to be vaccinated against COVID-19.

Stay safe,

Stanley Martin, MD, Division Chief, Infectious Disease

Email Version B2—Corrected: Reframing Risk (delayed control)

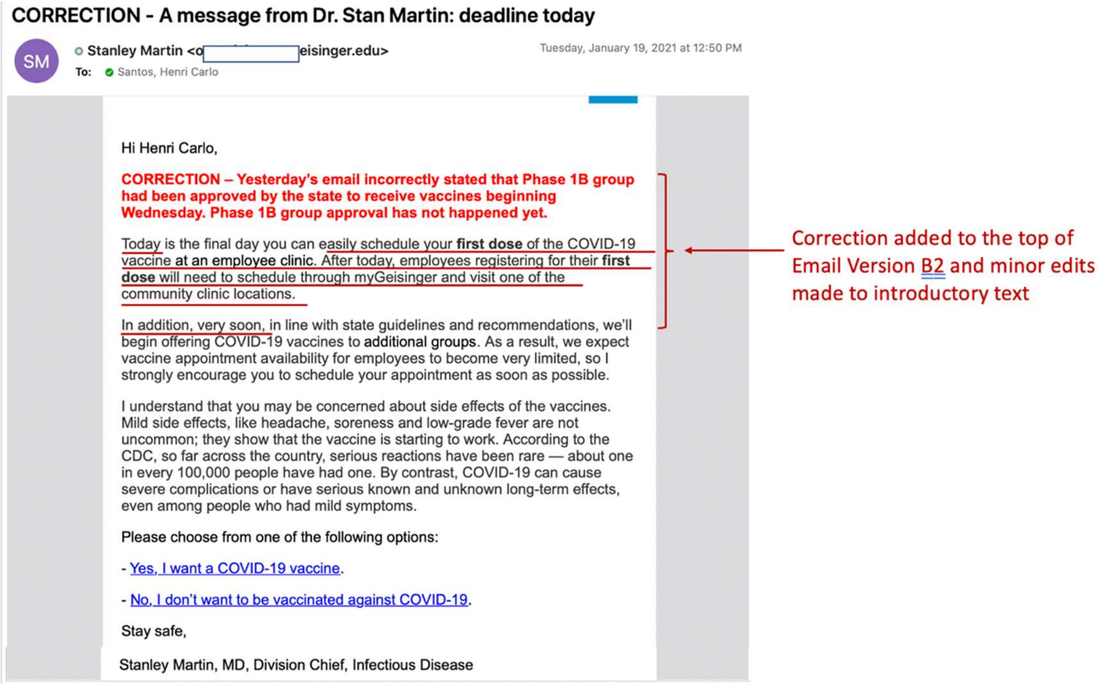

CORRECTION - A message from Dr. Stan Martin: deadline today

SM Stanley Martin <o[illegible]eisinger.edu> Tuesday, January 19, 2021 at 12:50 PM
To: Santos, Henri Carlo

Hi Henri Carlo,

CORRECTION – Yesterday's email incorrectly stated that Phase 1B group had been approved by the state to receive vaccines beginning Wednesday. Phase 1B group approval has not happened yet.

Today is the final day you can easily schedule your **first dose** of the COVID-19 vaccine **at an employee clinic**. After today, employees registering for their **first dose** will need to schedule through myGeisinger and visit one of the community clinic locations.

In addition, very soon, in line with state guidelines and recommendations, we'll begin offering COVID-19 vaccines to **additional groups**. As a result, we expect vaccine appointment availability for employees to become very limited, so I strongly encourage you to schedule your appointment as soon as possible.

I understand that you may be concerned about side effects of the vaccines. Mild side effects, like headache, soreness and low-grade fever are not uncommon; they show that the vaccine is starting to work. According to the CDC, so far across the country, serious reactions have been rare — about one in every 100,000 people have had one. By contrast, COVID-19 can cause severe complications or have serious known and unknown long-term effects, even among people who had mild symptoms.

Please choose from one of the following options:

- Yes, I want a COVID-19 vaccine.

- No, I don't want to be vaccinated against COVID-19.

Stay safe,

Stanley Martin, MD, Division Chief, Infectious Disease

© 2021 Santos HC et al. *JAMA Network Open.*

Survey Materials

What is the main reason you're declining to receive an FDA-authorized COVID-19 vaccine? (Your responses are anonymous and cannot be linked to your name or email.)

- Concerns about known side effects (e.g., headache, fatigue)
- Concerns about unknown risks
- I don't feel I need to be vaccinated; I'm not at high risk for infection
- I don't feel I need to be vaccinated; I'm not at high risk for serious complications from COVID if I am infected
- It depends on when it's offered to me; I'd like to wait to take it until I see how it goes with others
- It depends on which vaccine I'm offered
- I've had COVID so I don't think I need a vaccine
- Privacy concerns about Geisinger or state government tracking
- I don't trust the FDA approval process
- I am pregnant, breastfeeding or planning to become pregnant
- I have a preexisting medical condition that would prevent me from getting the vaccine
- I have allergies that could react to the vaccine
- Concerns about the vaccine's ingredients (e.g., mRNA)
- Concerns about the vaccine's efficacy
- I work out of state; I plan to receive a vaccine when one is available to me in my state
- Other ____

© 2021 Santos HC et al. *JAMA Network Open.*

eFigure. Study Flowchart

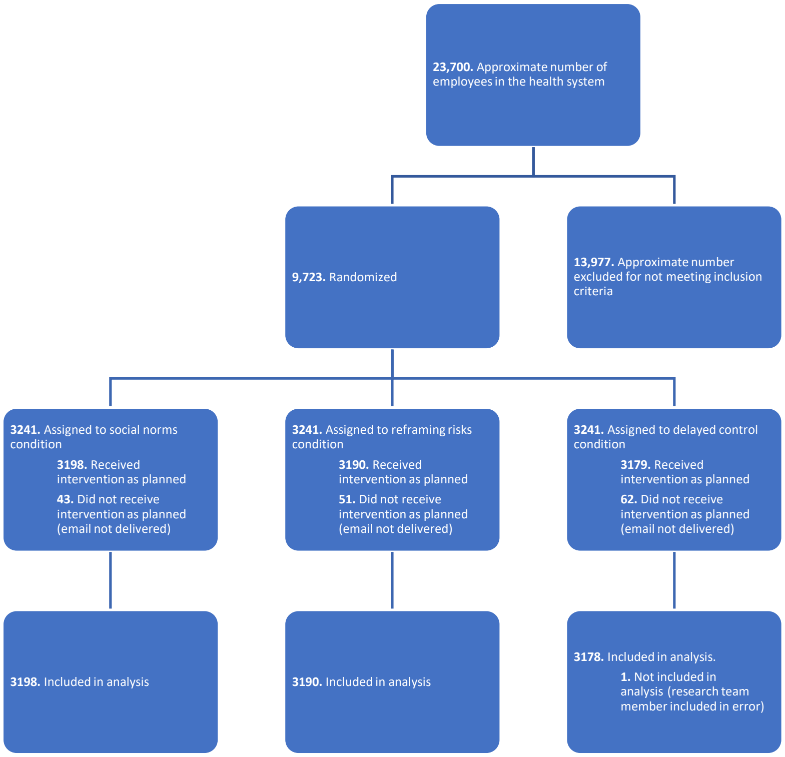

© 2021 Santos HC et al. *JAMA Network Open.*

eReferences.

1. Patel MS, Volpp KG, Small DS, et al. Using Active Choice Within the Electronic Health Record to Increase Influenza Vaccination Rates. *J Gen Intern Med*. 2017;32(7):790-795. doi:10.1007/s11606-017-4046-6
2. Bakr O, Afsar-Manesh N, Raja N, et al. Application of Behavioral Economics Principles Improves Participation in Mailed Outreach for Colorectal Cancer Screening. *Clin Transl Gastroenterol*. 2020;11(1):e00115. doi:10.14309/ctg.0000000000000115
3. Moehring A, Collis A, Garimella K, et al. Surfacing norms to increase vaccine acceptance. *psyArXiv*, https://psyarxiv.com/srv6t/. 2021.
4. Mheidly N, Fares J. Leveraging media and health communication strategies to overcome the COVID-19 infodemic. *J Public Health Policy*. 2020;41(4):410-420. doi:10.1057/s41271-020-00247-w
5. Tversky A, Kahneman D. The framing of decisions and the psychology of choice. *Science*. 1981;211(4481):453-458. doi:10.1126/science.7455683

© 2021 Santos HC et al. *JAMA Network Open.*
