# Supplementary material for: Effect of Targeted Behavioral Science Messages on COVID-19 Vaccination Registration Among Employees of a Large Health System: A Randomized Trial
Source: JAMA Netw Open. 2021 Jul 28;4(7):e2118702. doi: 10.1001/jamanetworkopen.2021.18702 (PMC8319759; doi:10.1001/jamanetworkopen.2021.18702)
Supplement: Supplement 2. — Trial Protocol [file jamanetwopen-e2118702-s002.pdf]

Sending Emails Designed with Behavioral Science to Increase COVID-19 Vaccination (NCT04728594)

Study Protocol with Statistical Analysis Plan

May 21, 2021

Study Protocol

Purpose

The purpose of the study was to evaluate, prospectively, the potential impact of different email message conditions (social norms, reframing risk) on registration for COVID-19 vaccination by Geisinger Health System employees who have not yet made an appointment.

Introduction

Laboratory studies of interventions to increase COVID-19 vaccination intentions and field tests of behavioral “nudges” to increase uptake of other vaccines suggest that behavioral science can contribute to reaching population immunity. The first opportunities to field test such interventions in the COVID-19 context are with healthcare workers (HCWs), who are among the first to be offered COVID-19 vaccines and are important ambassadors for COVID-19 vaccine acceptance in the general population. While most HCWs employed by a large Pennsylvania health system reported intentions to get vaccinated, many were hesitant due to concerns about vaccine side effects and unknown risks and wanted to wait and see how others fared with vaccination. That system’s initial communication strategy entailed sending 36 vaccine-related mass messages to all employees over five weeks (supplement). After this effort, around 60% had scheduled their vaccinations. We targeted the remainder, testing two direct emails designed with behaviorally-informed features to promote vaccination against a delayed control group.

Methods

Sample

Eligible employees were those without a COVID-19 appointment on record. Sample size was determined by the number of eligible employees (if our study had not been conducted, all would have received one email from the health system promoting vaccination).

Experimental and control conditions

All emails were designed to be a personal appeal from a medical expert and authority figure in the health system. The first paragraph of the emails emphasized the upcoming scarcity of the vaccines, and the options encouraged employees to make an active choice of whether to get the vaccine or not. Those who clicked a link to get the vaccine were automatically sent to a scheduling portal. Those who did not choose to get the vaccine were automatically sent to an online survey.

The online survey presented 16 reasons for COVID-19 vaccine hesitancy. After respondents selected their main reason for hesitancy, we presented explanations that aimed to address that particular concern. These explanations were largely drawn from the health system’s FAQ on the vaccines. After reading this explanation, respondents were given another chance to register for an appointment. The online survey was anonymous, so we could not link responses to experimental condition or actual registration for the vaccine.

Social norms. In the social norms condition, employees received an email from the health systems' division chief of infectious disease. The email used an action-oriented subject line, "Indicate your COVID-19 vaccine plan," and contained the following content:

Hi [first name],

Very soon, in line with state guidelines and recommendations, we'll be offering COVID-19 vaccines to all those who qualify as part of the state's Phase 1B groups, which is a large group of people. As a result, we expect vaccine appointment availability for employees to soon become very limited, so I strongly encourage you to schedule your appointment as soon as possible.

More than 11 million Americans have received a COVID-19 vaccine. This includes more than 14,000 of your Geisinger colleagues — including more than 80% of providers like me — and more are already scheduled.

Please choose from one of the following options:

- *Yes, I want a COVID-19 vaccine.*
- *No, I don't want to be vaccinated against COVID-19.*

Stay safe,

Dr. Stan Martin, Division Chief, Infectious Diseases

Reframing Risk. In the reframing risk condition, employees received an email from the health systems' division chief of infectious disease. The email used a matter-of-fact subject line: "A message from Dr. [first name, last name],"and contained the following content:

Hi [first name],

Very soon, in line with state guidelines and recommendations, we'll be offering COVID-19 vaccines to all those who qualify as part of the state's Phase 1B groups, which is a large group of people. As a result, we expect vaccine appointment availability for employees to soon become very limited, so I strongly encourage you to schedule your appointment as soon as possible.

I understand that you may be concerned about side effects of the vaccines. Mild side effects, like headache, soreness and low-grade fever are not uncommon; they show that the vaccine is starting to work. According to the CDC, so far across the country, serious reactions to the vaccine have been rare — about 1 in every 100,000 people have had one. By contrast, COVID-19 can cause severe complications or have serious known and unknown long-term effects, even among people who had mild symptoms.

Please choose from one of the following options:

- Yes, I want a COVID-19 vaccine.
- No, I don't want to be vaccinated against COVID-19.

Stay safe,

Dr. Stan Martin, Division Chief, Infectious Diseases

Delayed control. Participants in the delayed control group did not receive the emails until January 18, 2021. A data pull of the primary outcome was planned for January 18, 2021, but the data was not able to be reliably gathered at that time. Without any reliable data on the performance of the two groups from which to choose a "winning message," we decided to evenly randomize the delayed control group to both email versions. The emails sent to the delayed control group included "deadline tomorrow" at the end of the subject line and the first paragraph was revised to inform employees about the end of the employee-specific distribution of vaccines:

Tomorrow is the final day you can easily receive a COVID-19 vaccine as an employee. Starting Wednesday, in line with state guidelines and recommendations, we'll begin offering COVID-19 vaccines to all those who qualify as part of the state's Phase 1B groups, a large group of people. As a result, we expect vaccine appointment availability for employees to soon become very limited, so I strongly encourage you to schedule your appointment as soon as possible.

The following day a correction to the emails sent to the delayed control group was sent; it included "CORRECTION" at the start of the subject line and the following message at the start of the email in red and boldface (the correction did not change any information about the distribution plan or upcoming deadline):

CORRECTION – Yesterday's email incorrectly stated that Phase 1B group had been approved by the state to receive vaccines beginning Wednesday. Phase 1B group approval has not happened yet.

Outcome measures

The primary outcome measure was registration for a COVID-19 vaccination made through the employee scheduling portal. The secondary outcomes were whether the email was opened and whether the link in it was clicked. All measures were binary and measured by the time the delayed group was emailed (12:49 EST, January 18, 2021) and again by the time the link no longer sent respondents to the online registration portal (13:19 EST, January 19, 2021). The full study period, including primary and exploratory analysis of the outcomes four days after the intervention, was from 16:55 EST, January 15, 2021 to 13:19 EST, January 19, 2021.

Registration during the first three days was the ultimate outcome of interest, since it reflected the behavioral choice that could lead to vaccination, and by limiting analysis to this period we could measure the effect of the two email interventions relative to the control group.

In our ClinicalTrials.gov record, we preregistered that the study would examine data after two days. This was extended to about three days, which was the time when the delayed control group was emailed. This altered timing was dictated by when the Geisinger Marketing department was ready to send emails to the delayed control group. Data for email opens and link clicks, as well as registrations, opens, and link clicks were recorded and were uploaded on the Open Science Framework.

Statistical Analysis Plan

The researchers hypothesized that more people receiving either of the emails designed with behavioral science will register for COVID-19 vaccinations than those the delayed-contact control group.

Binary logistic generalized linear models (GLMs) were used to analyze registration, email open, and email link click rates as a function of experimental condition. The data was analyzed with logistic regression models with the control group as the reference group, to compare the two email conditions versus the control group. This set of analyses was only conducted for scheduling a vaccination appointment as opposed to email engagement outcomes (e.g., number of emails opened), which was not applicable for the control group. A second set of logistic regression models predicting scheduling a vaccination appointment and email engagement were run, comparing the two email conditions against each other. This comparison was exploratory since we had no reason to predict one message would be superior to the other.

Odds ratios (ORs) were calculated, along with asymptotic 95% confidence intervals (CIs); two-tailed p-values < 0.05 were used to determine statistical significance. Raw percentages with asymptotic 95% CIs were also presented in graphs. All analyses were conducted in R.
