# Supplementary material for: Effect of Targeted Behavioral Science Messages on COVID-19 Vaccination Registration Among Employees of a Large Health System: A Randomized Trial
Source: JAMA Netw Open. 2021 Jul 28;4(7):e2118702. doi: 10.1001/jamanetworkopen.2021.18702 (PMC8319759; doi:10.1001/jamanetworkopen.2021.18702)
Supplement: Supplement 3. — Data Sharing Statement [file jamanetwopen-e2118702-s003.pdf]

Data Sharing Statement

Santos. Effect of Targeted Behavioral Science Messages on COVID-19 Vaccination Registration Among Employees of a Large Health System. *JAMA Netw Open*. Published July 28, 2021.
doi:10.1001/jamanetworkopen.2021.18702

Data
Data available: Yes
Data types: Deidentified participant data, Data dictionary
How to access data: OSF, https://osf.io/qg5m6/
When available: With publication

Supporting Documents
Document types: Statistical/analytic code, Other (please specify)
Additional Information: Materials
How to access documents: OSF, https://osf.io/qg5m6/
When available: With publication

Additional Information
Who can access the data: Anyone requesting the data
Types of analyses: Any purpose
Mechanisms of data availability: Open access de-identified data, code, and materials-no fees, no permissions necessary.
Any additional restrictions: None
